# The effect of P2X7 antagonism on subcortical spread of optogenetically-triggered cortical spreading depression and neuroinflammation

**DOI:** 10.1186/s10194-024-01807-1

**Published:** 2024-07-24

**Authors:** Burak Uzay, Buket Donmez-Demir, Sinem Yilmaz Ozcan, Emine Eren Kocak, Muge Yemisci, Yasemin Gursoy Ozdemir, Turgay Dalkara, Hulya Karatas

**Affiliations:** 1 Institute of Neurological Sciences and Psychiatry, Hacettepe University, Sihhiye, Ankara, 06100 Türkiye; 2 Department of Psychiatry, Hacettepe University, Ankara, Türkiye; 3 Department of Neurology, Hacettepe University, Ankara, Türkiye; 4 Department of Pharmacology, Vanderbilt University, Nashville, TN USA; 5 School of Medicine, Koc University, Istanbul, Türkiye

**Keywords:** Migraine, CSD, Subcortical spread, Neuroinflammation, P2X7R

## Abstract

Migraine is a neurological disorder characterized by episodes of severe headache. Cortical spreading depression (CSD), the electrophysiological equivalent of migraine aura, results in opening of pannexin 1 megachannels that release ATP and triggers parenchymal neuroinflammatory signaling cascade in the cortex. Migraine symptoms suggesting subcortical dysfunction bring subcortical spread of CSD under the light. Here, we investigated the role of purinergic P2X7 receptors on the subcortical spread of CSD and its consequent neuroinflammation using a potent and selective P2X7R antagonist, JNJ-47965567. P2X7R antagonism had no effect on the CSD threshold and characteristics but increased the latency to hypothalamic voltage deflection following CSD suggesting that ATP acts as a mediator in the subcortical spread. P2X7R antagonism also prevented cortical and subcortical neuronal activation following CSD, revealed by bilateral decrease in c-fos positive neuron count, and halted CSD-induced neuroinflammation revealed by decreased neuronal HMGB1 release and decreased nuclear translocation of NF-kappa B-p65 in astrocytes. In conclusion, our data suggest that P2X7R plays a role in CSD-induced neuroinflammation, subcortical spread of CSD and CSD-induced neuronal activation hence can be a potential target.

**Supplementary Information:**

The online version contains supplementary material available at 10.1186/s10194-024-01807-1.

## Background

Migraine is a primary episodic headache disorder that affects 10–20% of the population and has a big negative impact on the daily functioning of patients [1]. The pathophysiology of migraine is yet to be fully elucidated but the *cortical spreading depression* (CSD) is known to have a vital role in the pathophysiology of migraine. CSD is the slowly spreading depolarizing electrical activity on the cortex and is the electrophysiological correlate of the migraine aura [2–4]. Previous research has showed that CSD results in opening of the neuronal pannexin-1 (Panx1) channels, release of proinflammatory cytokines and alarmins (such as HMGB1 and IL1β) in neurons and activation of the inflammatory cascade by translocation of p65 subunit of NFκB to the nucleus in the parenchymal astrocytes [5]. This parenchymal inflammation activates meningeal nociceptors via glia limitans and the trigeminovascular system causing headache [5]. Panx1 megachannels are known to be closely associated with purinergic P2X7 receptors (P2X7R) that are activated by extracellular ATP and that can further activate the inflammatory cascade, rendering them a worthy target in investigating the spread of the CSD and its consequent neuroinflammation [6]. A study showed that genetic loss of P2X7R as part of the P2X7/Panx1 pore suppresses spreading depolarization, its inflammatory effects and the trigeminovascular activation in wild-type rats and mice [7].

CSD spreads to subcortical structures, although it has been almost exclusively investigated as a cortical phenomenon [8–10]. A H_2_^15^O-PET study showed that hypothalamus, along with some other subcortical structures (PAG, Putamen, Caudate nucleus) get activated during a migraine attack [11]. This activation is thought to cause some migraine symptoms suggesting subcortical dysfunction including nausea, sensations of cold/hot, yawning [12]. It is not known whether the subcortical spread of CSD is the reason of these symptoms and whether purinergic system and P2X7 receptors plays a role in this spread.

The conventional CSD induction methods are invasive (electrical stimulation, topical KCl application, pin-prick) and are preceded by a craniotomy [13]. These methods can depolarize neurons but damage vascular structures and astrocytes, thus have non-specific effects. CSD can be triggered non-invasively using optogenetic methods without disrupting the integrity of the skull eliminating the non-specific effects of invasive methods. Therefore, optogenetic induction of CSD is highly useful in investigating CSD-induced neuroinflammation [14]. In this study, we investigated the role of P2X7 receptors on cortical and subcortical parenchymal neuroinflammation, by inducing CSD via optogenetic tools to mitigate any potential neuroinflammation that is side effect of the induction method. To investigate the role of P2X7 receptors on the subcortical spread of CSD, we used both optogenetic tools and the pin-prick method as obtaining subcortical electrophysiology recordings is already invasive. To block P2X7 receptors we used a potent, selective and brain-blood barrier (BBB) permeable P2X7R antagonist, JNJ-47965567 systemically [15]. Upon P2X7R antagonist administration, we observed an increase in the latency to subcortical voltage deflection following CSD, and a decrease in subcortical c-fos positive neuron count underscoring the role of P2X7 receptors in mediating the subcortical spread of CSD. Moreover, P2X7R antagonism prevented the CSD-induced neuroinflammation, suggesting its role in migraine pathophysiology.

## Results

### P2X7 antagonism doesn’t change optogenetic CSD threshold and CSD characteristics

**Fig. 1:**
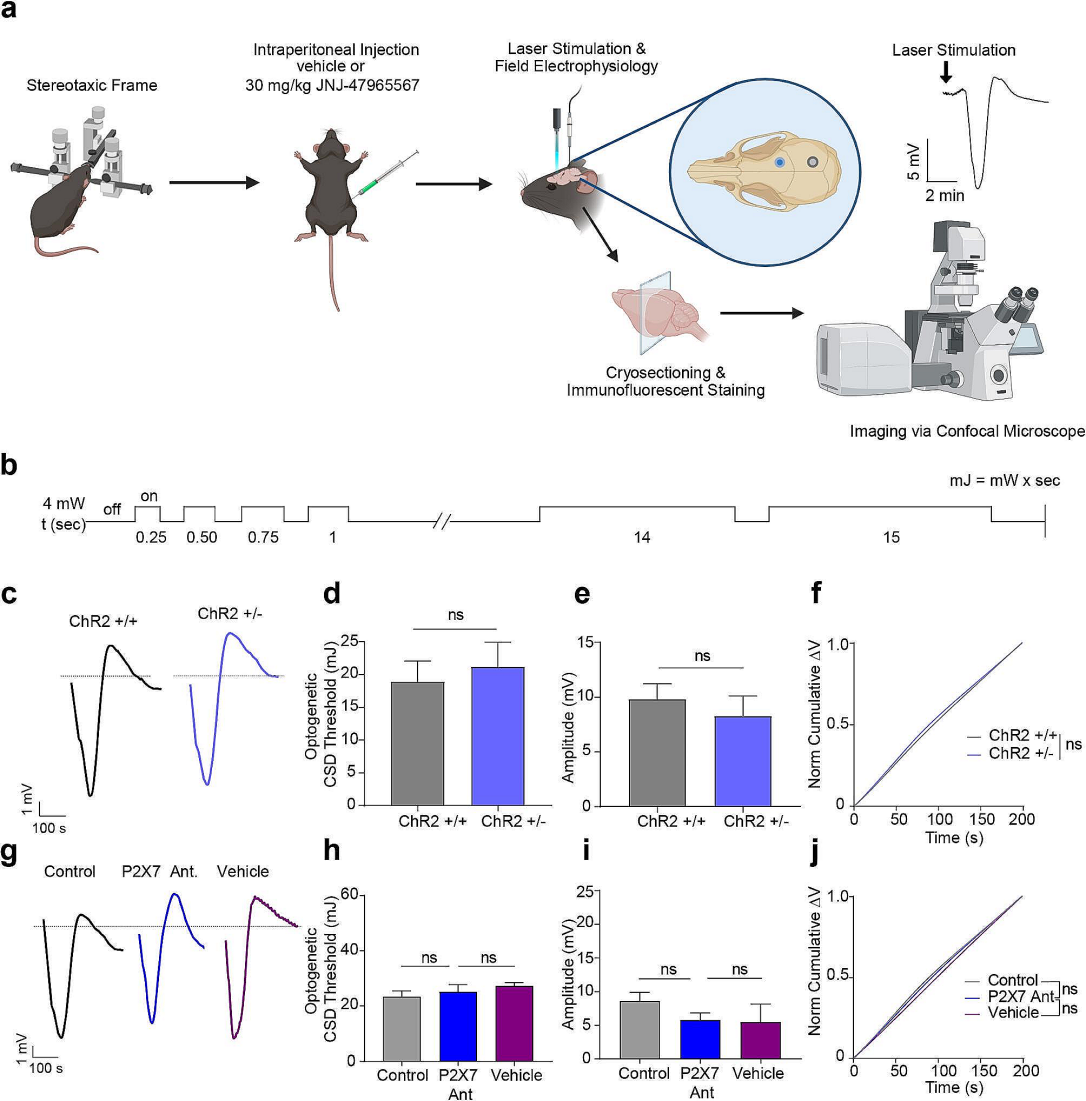
P2X7R antagonism doesn’t change optogenetic CSD threshold and CSD characteristics. **(a)** Schematic representation of the experimental protocol. **(b)** Schematic representation of optogenetic CSD threshold protocol. **(c)** Representative traces of optogenetically triggered CSD in homozygous (ChR2^+/+^) and heterozygous (ChR2^+/-^) mice. **(d)** Optogenetic CSD threshold (mJ) in ChR2^+/+^ (*n* = 9) and ChR2^+/-^ mice (*n* = 7). **(e)** CSD amplitude in ChR2^+/+^ and ChR2^+/-^ mice. **(f)** Normalized cumulative change in voltage during CSD in ChR2^+/+^ and ChR2^+/-^ mice. **(g)** Representative traces of optogenetically triggered CSD in control group and upon P2X7R antagonist or vehicle administration. **(h)** Optogenetic CSD threshold (mJ) in control group (*n* = 14) and upon P2X7R antagonist (*n* = 7) or vehicle administration (*n* = 6). **(i)** CSD amplitude in control group and upon P2X7R antagonist or vehicle administration. **(j)** Normalized cumulative change in voltage during CSD in control group and upon P2X7R antagonist or vehicle administration. CSD: Cortical Spreading Depression, ChR2: channelrhodopsin (ns: p > 0.05, *:p < 0.05, **:p < 0.01, ***:p < 0.001)

We used the CSD threshold protocol which is optimized for optogenetic tools in [14](Fig. 1a, b) and found that the average CSD threshold was 17.9 mJ (±2.3 S.E.M; *n* = 15). The minimum energy that could induce CSD was 6 mJ and the maximum was 28 mJ. CSD threshold or amplitude didn’t differ between homozygous (15.7 mJ ± 3 S.E.M; *n* = 9) and heterozygous (21.4 mJ ±3.9 S.E.M; *n* = 7) Thy1-ChR2 mice (*p* = 0.31) (Fig. 1c-f). Neither P2X7R antagonist (*n* = 7) nor the vehicle administration (*n* = 6)(30% cyclodextrin-sulfobutyl ether sodium salt; hereby it will be referred as the ‘vehicle’) had an effect on optogenetically induced CSD threshold or amplitude (*p* > 0.05)(Fig. 1g-j).

We explored the impact of P2X7 receptor antagonism on CSD parameters and velocity by conducting experiments on Balb/C mice since C57BL/6 mice are known for defective P2X7 receptors due to a natural P451L mutation [16]. We administered the P2X7R antagonist or its vehicle to groups of Balb/C mice and induced CSD through pin-prick after a 15-minute P2X7R antagonist application. We did not observe any significant differences in CSD speed, total duration, or half maximum duration between the groups (Supplementary Fig. 1a-c). Our findings on the effects of P2X7R antagonism did not differ between Balb/C and C57BL/6 mice (Supplementary Fig. 1d).

Migraine is more common in women, and sexual dimorphism has been observed in experimental models of migraine [17]. Estrogen can alter CSD kinetics and induction thresholds in female rodents. To test whether sex differences account for any possible changes in CSD kinetics following P2X7R antagonist administration, we conducted pinprick-induced CSD experiments in both female (*n* = 4) and male (*n* = 2) using Balb/c mice and found no significant differences in CSD characteristics between male and female groups (*p* > 0.1, Supplemantary Fig. 1e). We also performed optogenetic CSD inductions in both female (*n* = 4) and male (*n* = 5) Thy1-ChR2 mice, and again found no significant differences of CSD characteristics among the groups (*p* > 0.1, Supplementary Fig. 1f-g).

### Subcortical electrophysiological recordings show voltage deflections following CSD

**Fig. 2:**
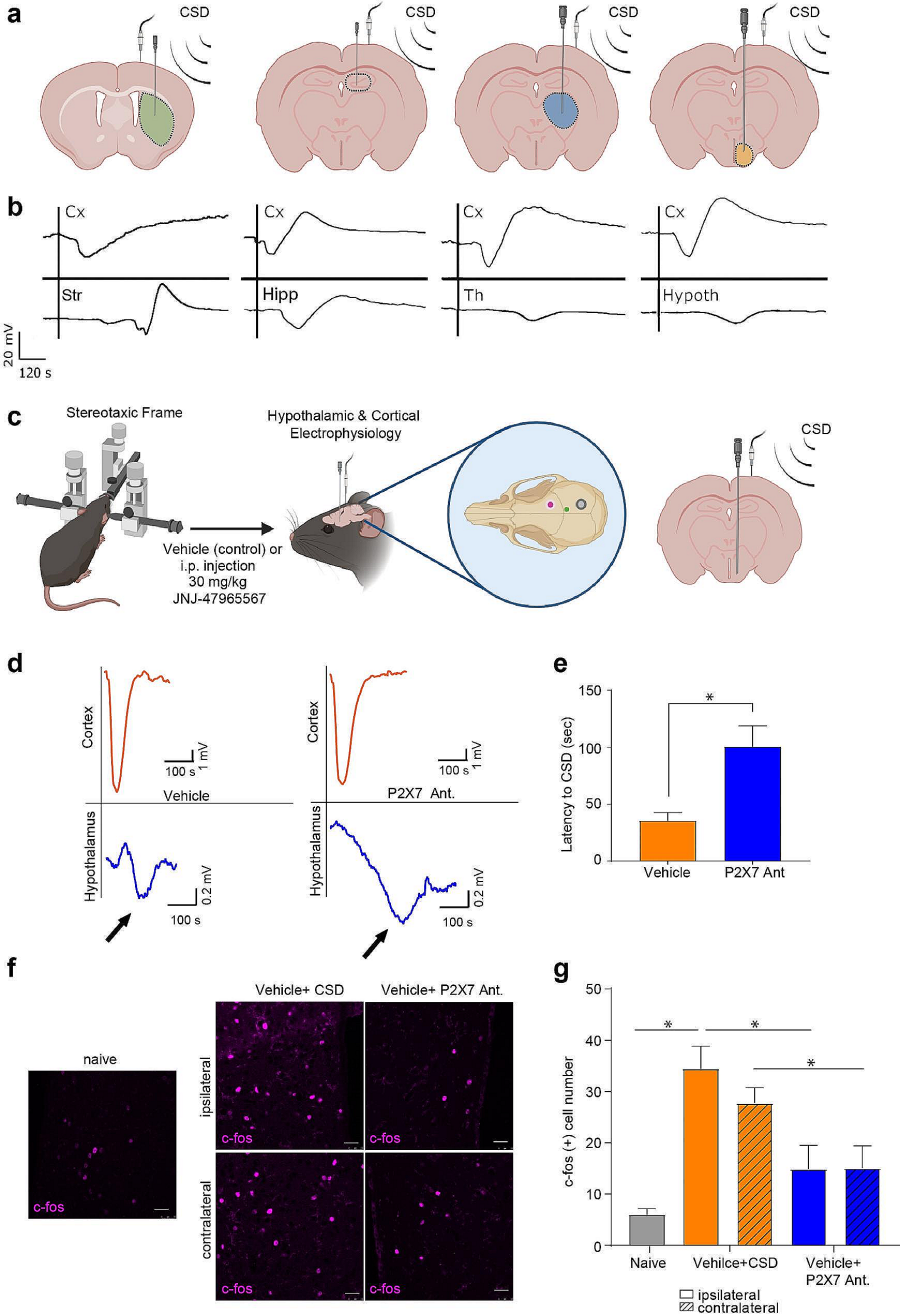
Hypothalamic voltage deflection following CSD happens under P2X7R antagonism with an increase in latency to CSD. **(a)** Schematic representation of cortical and subcortical electrophysiology. **(b)** Representative traces of cortical (Cx) and subcortical recordings from the striatum (Str), hippocampus (Hc), thalamus (Th) and hypothalamus (Hypoth). **(c)** Schematic representation of cortical and hypothalamic electrophysiology experimental protocol (green dot: insertion point of tungsten electrode for hypothalamic recordings, pink dot: pin-prick site, grey dot: surface electrode). **(d)** Representative traces of cortical and hypothalamic recordings in control group or upon P2X7R antagonist administration. **(e)** Hypothalamic voltage deflection latency to CSD in control group and upon P2X7R antagonist administration (*n* = 4/group). **(f)** Representative images of hypothalamic c-fos immunofluorescent staining in naïve mice, after CSD induction and upon P2X7R antagonist administration. *scale bar:25 µm* (**g**) c-fos positive cell numbers in hypothalamus of naïve mice, after CSD induction and upon P2X7R antagonist administration (*n* = 5/group). CSD: Cortical Spreading Depression (ns: p > 0.05, *:p < 0.05, **:p < 0.01, ***:p < 0.001)

We first performed subcortical electrophysiological recordings from different subcortical structures (striatum, hippocampus, thalamus and hypothalamus) simultaneously with cortical recordings. Each stimulus elicited spreading depolarization in the recorded subcortical structures (striatum, hippocampus, thalamus and hypothalamus) with varying latencies to CSD in Thy1-ChR2 (optogenetic CSD induction), Swiss albino and C57BL6 mice (pinprick CSD induction)(Fig. 2a-b).

### P2X7 antagonism increases the latency of hypothalamic voltage deflection following CSD

Hypothalamic electrophysiological recordings showed a voltage deflection followed by CSD with an average amplitude of 2.61 mV (± 0.76 S.E.M; *n* = 4) and with an average delay of 35.5 s (± 7.24 S.E.M; *n* = 4). Following P2X7R antagonist administration, hypothalamic recordings showed a voltage deflection with an amplitude of 1.33 mV (± 0.78 S.E.M; *n* = 4) and with a latency of 100.5 s (± 18.1 S.E.M; *n* = 4). P2X7R antagonism significantly increased the latency of hypothalamic voltage deflection following CSD (*p* = 0.01) (Fig. 2d-e).

We hypothesized that the prolonged latency to subcortical voltage deflection following CSD could be due to inhibited CSD propagation. To test if the propagation speed of CSD in the cortex is affected by P2X7R antagonism, we performed CSD induction experiments using double electrodes with Thy1-ChR2 mice. Our analysis revealed that there was no significant difference in CSD speed between the vehicle-treated group (*n* = 2) and the group injected with the P2X7R antagonist (*n* = 3)(Supplementary Fig. 1h). Despite the significantly prolonged latency to subcortical voltage deflection following CSD after P2X7R antagonist administration, our findings do not indicate a difference in the propagation speed of CSD in the cortex. Therefore CSD induced parenchymal neuroinflammatory signaling may differ from the electrophysiological spread of CSD. This was also the case in our previous report suggesting that Panx1 blockage suppressed the neuroinflammatory reaction after CSD but not the CSD generation or electrophysiological characteristics [5].

### CSD results in an increased neuronal activity in hypothalamus

We assessed hypothalamic activation following CSD, by counting hypothalamic c-fos positive neurons. Following CSD there was a significant increase in total number of c-fos positive neurons bilaterally in the hypothalamus (*p* = 0.04). This increase in c-fos positivity was prevented by P2X7R antagonist administration (*p* = 0.04) (Fig. 2f-g). In none of the groups, c-fos positive cell number differs between the two hemispheres (*p* > 0.99; *n* = 5–8/group). To test whether P2X7R antagonism affects c-fos positive neuron number in other brain structures following CSD, we took additional images from the cortex and the thalamus. We found that P2X7R antagonist administration preceding CSD induction results in a significant decrease of c-fos positive cell number, not only in the ipsilateral but also in the contralateral cortex and thalamus (Supplementary Fig. 2).

### P2X7 antagonism halts neuroinflammation following CSD

**Fig. 3:**
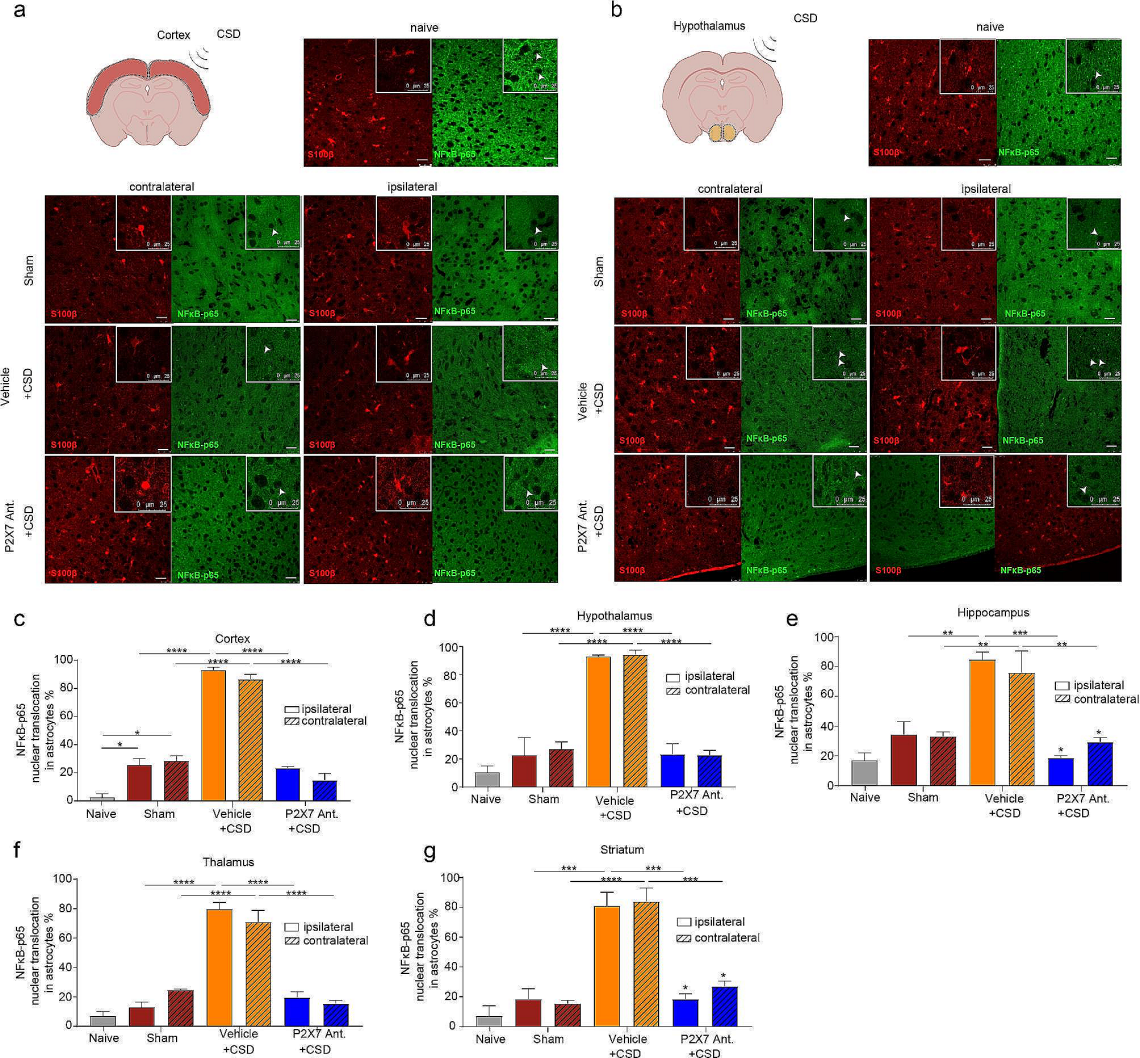
P2X7R antagonism prevents nuclear translocation of NFκB-p65 in astrocytes following CSD. (**a**) Representative images of cortical NFκB-p65 and S100β immunofluorescent co-staining in naïve, sham mice, and in mice following optogenetic CSD induction with or without P2X7R antagonist administration. *scale bar:25 µm* (**b**) Representative images of hypothalamic NFκB-p65 and S100β immunofluorescent co-staining. *scale bar:25 µm* (**c**) Percentage of nuclear translocation of NFκB-p65 in S100β-positive astrocytes in cortex in naïve, sham mice, and in mice following optogenetic CSD induction with or without P2X7R antagonist administration (*n* = 5/group). (**d**) Percentage of nuclear translocation of NFκB-p65 in S100β-positive astrocytes in hypothalamus (*n* = 5/group). (**e**) Percentage of nuclear translocation of NFκB-p65 in S100β-positive astrocytes in thalamus (*n* = 5/group). (**f**) Percentage of nuclear translocation of NFκB-p65 in S100β-positive astrocytes in hippocampus (*n* = 3/group). (**g**) Percentage of nuclear translocation of NFκB-p65 in S100β-positive astrocytes in striatum (n = 5/group). CSD: Cortical Spreading Depression (ns: p > 0.05, *:p < 0.05, **:p < 0.01, ***:p < 0.001)

**Fig. 4:**
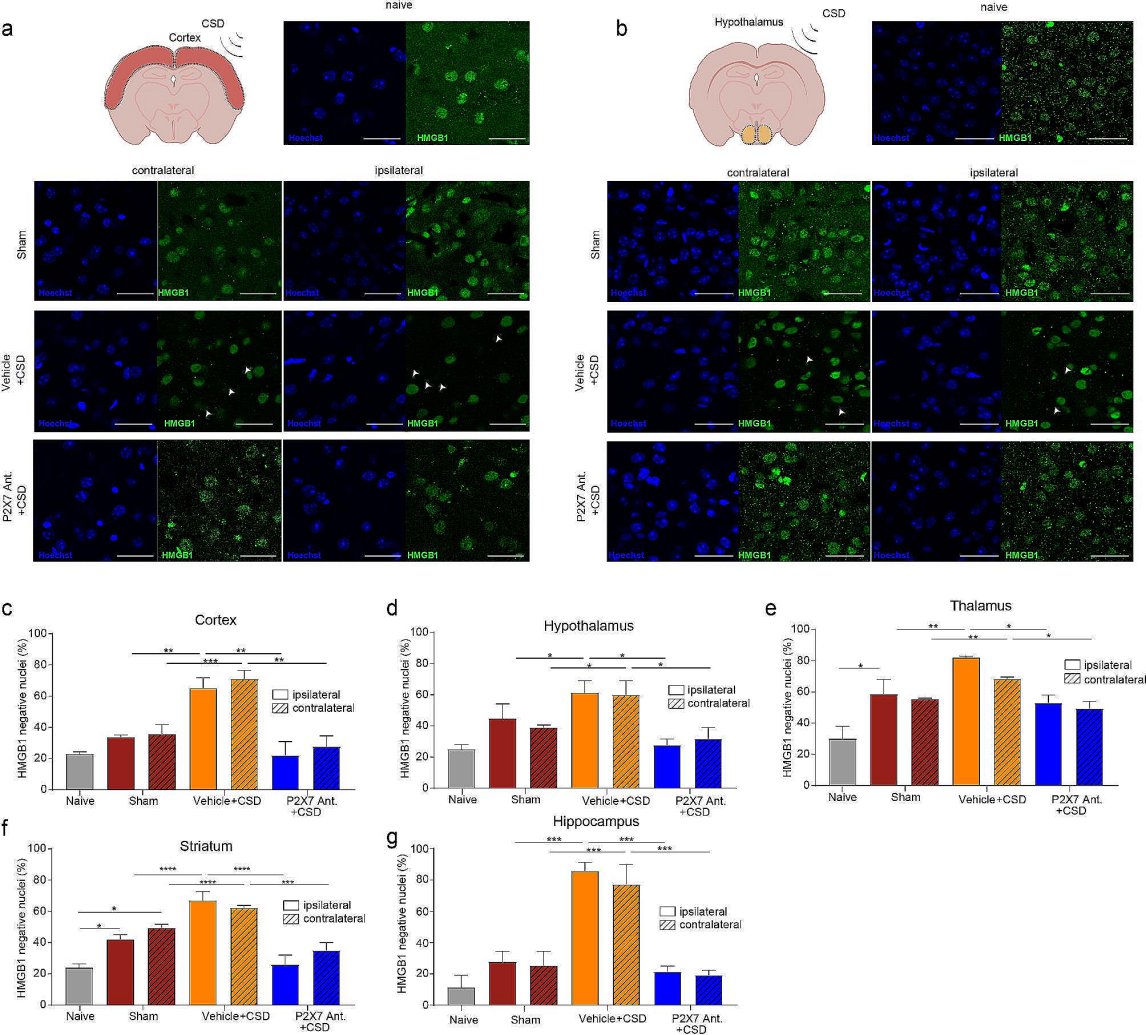
P2X7R antagonism prevents HMGB1 release following CSD. (**a**) Representative images of cortical HMGB1 immunofluorescent staining in naïve, sham mice, and in mice following optogenetic CSD induction with or without P2X7R antagonist administration. *scale bar:25 µm* (**b**) Representative images of hypothalamic HMGB1 immunofluorescent staining. *scale bar:25 µm* (**c**) Percentage of cortical HMGB1 release in naïve, sham mice, and in mice following optogenetic CSD induction with or without P2X7R antagonist administration (*n* = 5/group). (**d**) Percentage of hypothalamic HMGB1 release (*n* = 5/group). (**e**) Percentage of thalamic HMGB1 release (*n* = 5/group). (**f**) Percentage of striatal HMGB1 release (*n* = 3/group). (**g**) Percentage of hippocampal HMGB1 release (n = 3/group). CSD: Cortical Spreading Depression (ns: p > 0.05, *:p < 0.05, **:p < 0.01, ***:p < 0.001)

Following CSD, in the cortical and the subcortical structures (striatum, hippocampus, thalamus and hypothalamus), we observed an increase in the nuclear translocation of NFκB -p65 in S100β positive astrocytes and a decrease in neuronal HMGB1 positivity (HMGB1 release) supporting previous studies [5] (*p* < 0.001; *n* = 5/group)(Figs. 3a-g and 4a-g; Supplemental Fig. 1a-c, Supplemental Fig. 2a-c). P2X7R antagonism inhibited the translocation of NFκB-p65 to the nucleus of astrocytes and prevented HMGB1 release from neurons significantly thus inhibiting the neuroinflammation in both hemispheres (*p* < 0.001, *p* < 0.05; *n* = 5/group) (Figs. 3c-g and 4c-g; Supplemental Fig. 1a-c; Supplemental Fig. 2a-c). There was no difference in NFκB-p65 nuclear translocation or in HMGB1 release between the two hemispheres (*p* = 0.98).

### P2X7 receptor signal is increased in both cortical and subcortical structures following CSD and is colocalized to neurons

**Fig. 5:**
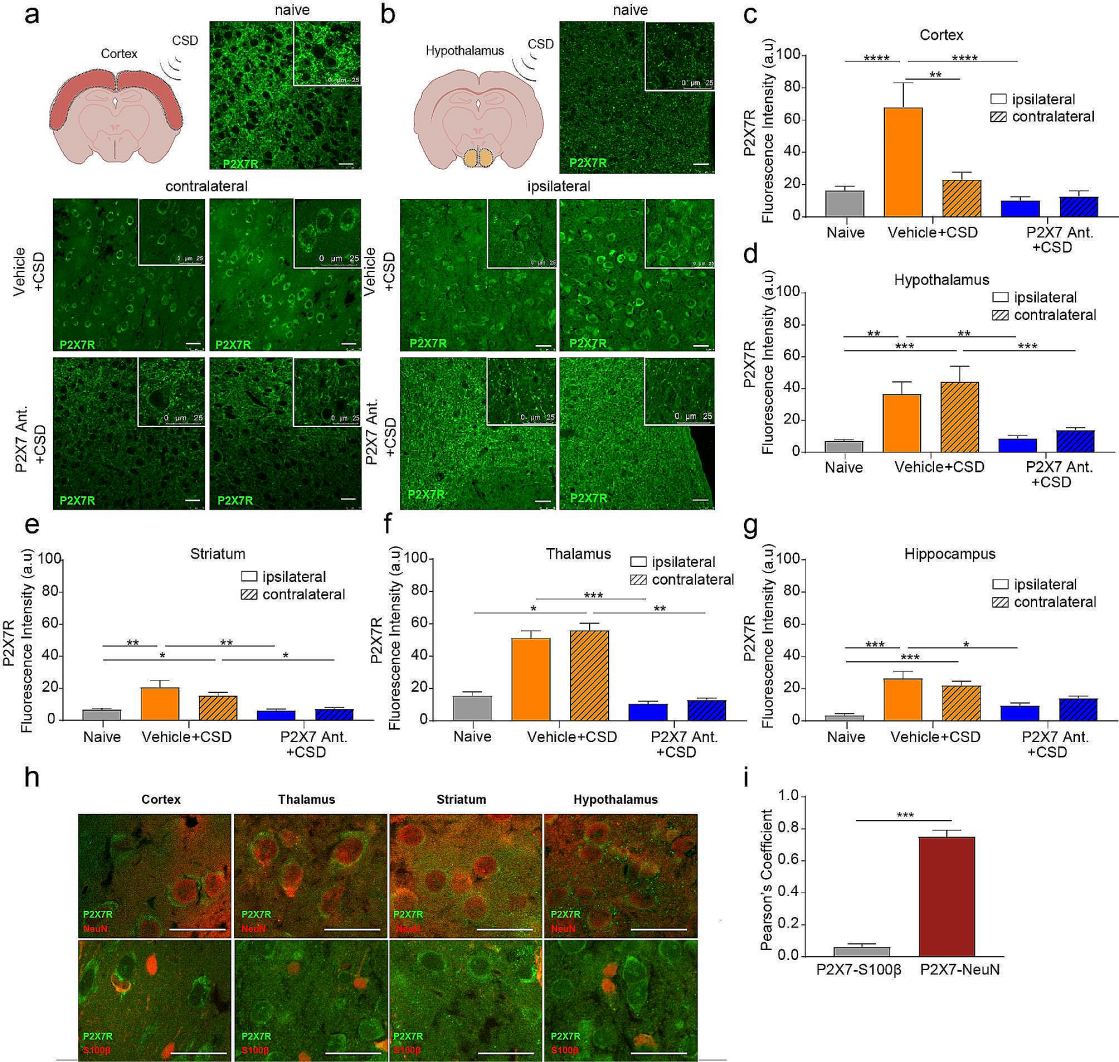
P2X7R signal is increased in cortical and subcortical structures that localizes to neurons and is prevented by P2X7R antagonism. (**a)** Representative images of cortical P2X7R immunofluorescent staining in naïve mice and in mice following optogenetic CSD induction with or without P2X7R antagonist administration. *scale bar:25 µm* (**b**) Representative images of hypothalamic P2X7R immunofluorescent staining. *scale bar:25 µm* (**c**) Fluorescence intensity of cortical P2X7R signal in naïve mice and in mice following optogenetic CSD induction with or without P2X7R antagonist administration (*n* = 5/group). (**d**) Fluorescence intensity of hypothalamic P2X7R signal (*n* = 5/group). (**e**) Fluorescence intensity of striatal P2X7R signal (*n* = 5/group). (**f**) Fluorescence intensity of thalamic P2X7R signal (*n* = 3/group). (**g**) Fluorescence intensity of hippocampal P2X7R signal (*n* = 5/group). (**h**) Representative images of P2X7R signal following CSD co-stained with either NeuN or S100β. (**i**) Pearson coefficient of P2X7R- S100β or P2X7R-NeuN colocalization (*n* = 3/group). (ns:p > 0.05, *:p < 0.05, **:p < 0.01, ***:p < 0.001)

In the naïve mice, on which neither interventions nor CSD induction was performed, we observed a membranous labeling of P2X7R whereas following CSD, there was a substantial increase in cytoplasmic signal. This increase in P2X7R signal was prominent in both hemispheres and the increase in the P2X7R signal fluorescence intensity was significant in cortex, striatum, thalamus, hypothalamus and hippocampus (*p* < 0.001, *p* = 0.02, *p* = 0.04, *p* = 0.004, *p* = 0.0003, respectively; *n* = 3/group) (Fig. 5a-g; Supplemental Fig. 3a-c). Between the two hemispheres in the subcortical structures the increase in P2X7R signal was not different however in the cortex, this increase was more substantial in the ipsilateral hemisphere than the contralateral one (*p* = 0.004). P2X7R antagonist application prevented this P2X7R signal intensity increase following CSD, in all of the investigated brain regions, cortex (*p* < 0.001), striatum (*p* = 0.003), thalamus (*p* = 0.009), hypothalamus (*p* = 0.004) and hippocampus (*p* = 0.004). Following P2X7R antagonist administration, the decrease in P2X7R signal intensity was bilateral in both hemispheres (Fig. 5a-g; Supplemental Fig. 3a-c).

We co-stained these sections with P2X7R and either NeuN (neuron marker) or S100β (astrocyte marker) to determine the cell type in which P2X7R signal increase takes place (Fig. 5h). Cytoplasmic P2X7 signal colocalized to NeuN significantly (*p* = 0.03; *n* = 3/group) (Fig. 5i).

## Discussion

Migraine is an episodic headache disorder and its pathophysiology is yet to be fully elucidated [1]. CSD is the electrophysiological equivalent of migraine aura which results in opening of Panx1 megachannels, neuronal HMGB1 release and induction of neuroinflammatory cascades in astrocytes that eventually result in neurogenic inflammation, trigeminal activation and headache [5]. CSD was long studied as a cortical phenomenon. Studies across various animal models, including rats, guinea pigs, cats, and monkeys, have consistently demonstrated the occurrence of CSD waves in both cortical and subcortical structures. Research dating back to the 1960s has extensively explored the propagation of CSD from the cortex to subcortical tissues [8, 18, 19]. Electrophysiological and biochemical analyses, alongside magnetic resonance imaging (MRI) studies, have corroborated these effects in subcortical regions beyond the neocortex [20]. In 2005, Henning et al. utilized manganese-enhanced MRI (MEMRI) to visualize CSD that is triggered by topical potassium chloride application in rats and observed signal increase which extends to various cortical regions such as the retrosplenial granular cortex, frontal cortex, piriform cortex, and perirhinal cortex accompanied by heightened signals from various subcortical structures including the CA 1–3 segment of the hippocampus, subiculum, dentate gyrus, thalamic nucleus, superior and inferior colliculus, and geniculate nucleus [20]. During a migraine attack, many symptoms could be explained by the subcortical spread of the CSD or by possible de novo spreading depressions in these subcortical structures [9]. Among these symptoms, alterations in the consciousness can be explained by thalamic spreading depression; alterations in the locomotion can be explained by striatal spreading depression; dysphoria, yawning and fluid retention can be consequences of hypothalamic spreading depression [9, 21, 22] The autonomic symptoms which are more pronounced in the prodromal phase of a migraine attack, could be an effect of subcortical spreading depressions causing hypothalamic dysfunction [23]. Previously, the subcortical spread of the CSD was investigated using two different strains of Familial Hemiplegic Migraine 1 (FHM1) transgenic mice. These mice have mutations in their CACNA1A gene resulting in mutant P/Q-type voltage gated calcium channels, R192Q or S218L missense mutations, respectively. These two mutants demonstrate different levels of cortical hyperexcitability and phenotypical severity (more severe in S218L mice), and authors found that the level of subcortical spread in these mice was correlated with the phenotypical severity of their mutation [9]. A further MRI study using S218L FHM1 mice confirmed that the CSD spreads from cortex to striatum and from hippocampus to thalamus [10]. The difference in the level of subcortical spread between these two different transgenic strains (R192Q and S218L) is explained by the differential increase in cortical hyperexcitability which overcomes the barriers such as low neuronal densities or white matter, that potentially halt CSD’s spread [9]. In S218L FHM1 mice, subcortical spread of CSD could be prevented by intraperitoneal guanosine application [9], which stimulates astrocytic glutamate reuptake, providing further evidence that extracellular glutamate, K^+^ and autacoid factors aid in the subcortical spread of CSD. In our study, we recorded waves of spreading depression in different subcortical brain areas including striatum, hippocampus, thalamus and hypothalamus after a single cortical CSD induction. This phenomenon has been noticed since early in vivo studies, however, the reported degree of penetration was variable and depended on the anesthetic that is used, in addition to other experimental parameters such as the mode of CSD induction and the method of detection [8, 24–26]. The anesthesia type emerges as a potential factor contributing to discrepancies in literature findings regarding the subcortical spread of CSD. We observed an extensive subcortical spread when urethane, an anesthetic that minimally suppresses cortical excitability [27], hence is accepted as the optimal general anesthetic to study CSD, was used. Our simultaneous electrophysiological recordings showed that the CSD spreads to subcortical areas with a considerable delay, supporting the original idea that it spreads via grey matter continuity [8, 19] rather than axonal conduction, which is much faster. These findings further raise the question if the delay of the subcortical spreading depression in regard to CSD could be affected by various factors including different extracellular mediators, extracellular ions (Ca^2+^, K^+^ etc.), in addition to different neuronal subtypes that depolarize and different neurotransmitters that are released upon depolarization of subcortical structures. The modulation of subcortical spread in distinct subcortical structures should be addressed by future studies.

CSD opens neuronal Panx1 megachannels and causes an increase in extracellular ATP concentration [5, 7]. Released ATP can potentially be an autacoid factor that aids in the subcortical spread of the CSD as evidence supports functional coupling of Panx1 and P2X7 receptors [7, 28]. Here, we used a potent and selective, BBB permeable P2X7R antagonist, JNJ-47965567, to investigate the effects of P2X7R antagonism on CSD’s subcortical spread [15]. We did not use any genetic manipulation (i.e. siRNA or shRNA) against P2X7 receptors, to avoid potential genetic compensation by other purinergic receptors. P2X7R antagonist administration didn’t change CSD characteristics or CSD threshold in line with the literature [7]. Chen et al. showed that selective pharmacological inhibition of the P2X7 channel did not affect spreading depolarization threshold or frequency [7]. However, systemic or topical administration of A-438079 and BBG which block P2X7/Panx1 complex, was found to increase the CSD threshold [7]. This suggests that Panx1 megachannels might serve as an important target in the induction and the spread of the CSD as the crucial step before P2X7 receptor activation [5]. It is also well known that purinergic receptors form heteromeric complexes and besides P2X7 receptor, there are other purinergic receptors including P2X2, P2X4 that could response to extracellular ATP [29–33]. The reason why P2X7R antagonism did not have an effect on the CSD threshold might be due to the genetic redundancy elicited by other purinergic receptors in response to an increase in extracellular ATP. Another possible reason may be the different characteristics of CSD induced parenchymal neuroinflammatory signaling and the electrophysiological spread of CSD. This was also the case in our previous report suggesting that Panx1 blockage suppressed the neuroinflammatory reaction after CSD but not the CSD generation or electrophysiological characteristics [5]. P2X7R antagonism, however, increased the latency of hypothalamic voltage deflection following CSD. This voltage deflection that we observe at hypothalamic recordings could also be a reflection of the spread of the CSD to amygdala or to the piriformis cortex on the electrode. Regardless, the significant increase in the latency suggests that extracellular ATP acts as a mediator in the subcortical spread of CSD. This finding supports previous studies that show modulation of neuronal excitability through P2X7R activation [34, 35].

In the literature, CSD is conventionally induced by electrical stimulation, topical KCl application or pin-prick, which have non-specific glial and vascular effects [14]. Following the utilization of optogenetics, CSD was successfully induced without disrupting integrity of the skull [14]. This method is particularly useful to study inflammatory pathways associated with CSD, discarding the non-specific inflammation associated with craniotomy. Here, we used Thy1-ChR2 transgenic mice and stimulated the motor cortex via a blue laser (450 nm) to induce CSD. The optogenetic CSD threshold along with other CSD characteristics (amplitude and normalized cumulative change in voltage) were determined and didn’t differ between homozygous and heterozygous transgenic animals which is in line with the literature [36]. Besides, we recorded spreading depression in subcortical regions in C57BL6 and Swiss albino female and male mice by pinprick. These results suggest that induction method, gender or mice strain have no effect on CSD or its subcortical spread.

In various studies, c-fos (an early neuronal activation marker) is used as an indicator of CSD spread to the subcortical structures [9]. In the wild-type mice and R192Q and S218L-mutant FHM1 mice there was an increase in cortical c-fos positivity and in S218L mice there was an increase in c-fos positive neurons unilaterally in hippocampus, bilaterally in thalamic and lateral hypothalamic nuclei [9]. In another study, following CSD induction in freely-moving rats, abundant c-fos positivity was observed in the thalamic reticular nuclei and magnocellular area of hypothalamus [37]. Same study also showed that anesthesia application (thiopental, chloral hydrate) decreased aforementioned c-fos positivity, further suggesting that anesthesia alone can halt subcortical spread of CSD [37]. In our study, we found a bilateral increase in the c-fos positive neuron number in hypothalamus following CSD. P2X7R antagonist administration, however, prevented this increase in the c-fos positive neuron number, pointing out the role of P2X7 receptors in the hypothalamic neuronal activation following CSD. We observed the similar effect in the thalamus and the cortex. Our simultaneous hypothalamic and surface electrophysiology recordings show that P2X7R antagonist administration does not prevent subcortical spread to hypothalamus; however, it can delay the spread. Although CSD reaches hypothalamus, depolarization does not result in a significant increase in c-fos positive cells. This might stem from a number of factors including altered excitability of hypothalamic neurons and a potential decrease in released neuroinflammatory mediators following the administration of P2X7R antagonist.

CSD opens neuronal Panx1 megachannels and induces neuroinflammation [5]. Opening of Panx1 megachannels results in an increase in extracellular ATP concentration [7]. Panx1 megachannels are also known to be in close relation to the P2X7 receptors on the membrane and that P2X7 receptor activation triggers the inflammatory pathways rendering P2X7 as a potential target in neuroinflammation [38, 39]. In our study, P2X7R antagonism prevented the nuclear translocation of NFκB-p65 and HMGB1 release in cortex, thalamus, striatum, hippocampus and hypothalamus following optogenetically-induced CSD. This finding indicates that the opening of the Panx1 megachannels following CSD induces inflammation via a P2X7 receptor-mediated mechanism triggered by increased extracellular ATP. In another study, supporting our finding, application of P2X7/Panx1 complex blockers, A-438079 and BBG preceded by CSD was shown to decrease cortical IL1β mRNA levels [7]. The release of the HMGB1 starts a cascade of event both in the neighboring neurons (opening of Panx1 channels) and in the neighboring glia, which are constantly surveilling the environment and functionally sensitive to stress signals. In our study, we specifically examined NFκB-p65 and HMGB1 due to their well-established roles in neuroinflammation [40], particularly following CSD [41, 42]. Based on our findings, we believe that extracellular ATP, released by neurons, bind to the P2X7 receptors in the neighboring neurons, resulting in increase in cytokines and chemokines that in turn more robustly induce neuroinflammation in the glia. Extracellular ATP could potentially be binding to the P2X7 receptors on astrocytes, which warrants further investigation in future studies. While our study sheds light on the involvement of P2X7 receptors in CSD-induced neuroinflammation, it is important to acknowledge that there may be other neuroinflammatory markers and downstream molecules that warrant investigation in future research. Understanding the broader spectrum of neuroinflammatory markers and their relationships with purinergic signaling will be essential for gaining a comprehensive understanding of the mechanisms underlying CSD-induced neuroinflammation.

HMGB1 release occurs due to neuronal stress, which can set off subsequent reactions leading to neuroinflammation, though it is not a direct sign of it [5, 41]. Our surgical approach to induce CSD, involves delicately thinning a portion of the posterior parietal cortex using a drill for electrode insertion, while ensuring the skull’s integrity remains intact. Our experiments reveal that even minor manipulations of the skull, like drilling, can trigger neuronal stress responses in specific brain regions like the thalamus and the striatum, resulting in HMGB1 release. This phenomenon is supported by a recent publication that shows that drilling of the skull, without induction of CSD, increases the HMGB1 release in the hemisphere where drilling occurred as well as in the striatum [41].

We did not only see the effects of P2X7R antagonism on neuroinflammation in the ipsilateral hemisphere where CSD was induced but also in the contralateral hemisphere. Historically, CSD was thought to affect only a single hemisphere where the contralateral hemisphere was used as the control [13, 43]. However, a recent study showed that the effect of CSD-induced neuroinflammation is bilateral in wild-type and FHM1 mutant mice brains [41]. The contralateral neuroinflammatory responses could be explained by intense axonal volleys, which typically precede the depression of electrical activity during CSD initiation. These volleys propagate to the contralateral cortex via the corpus callosum and likely relay to downstream subcortical regions. Intense excitatory firing, akin to epileptiform discharges, can activate Panx1 megachannels, thus triggering downstream pathways secondary to NMDA receptor overactivation [41, 44]. Supporting this notion, authors successfully attenuate the neuroinflammatory profile in the contralateral cortex and striatum by locally inhibiting NMDA receptors with a use-dependent NMDA antagonist, MK801 in the contralateral cortex [41]. Human PET studies support these findings showing that the neuroinflammation following CSD is multiregional and can take place bilaterally [4]. A recent PET-MRI study confirmed this finding which was conducted with patients that had at least 1 migraine attack with aura and healthy controls, used a (^11^C)PBR28 radio ligand that binds to a glial marker 18 kDa translocator protein which increases during neuroinflammation, showed an increase in inflammatory signal in cortical and subcortical structures bilaterally in migraine patients and the signal intensity was found to correlate with the number of migraine attacks [45].

P2X7 receptors play an important role in the pathophysiology of various neuropsychiatric diseases and it has been long debated whether neuronal P2X7 receptors existed, if neuronal or glial P2X7R play a role in certain neurological and psychiatric disease pathophysiologies [46, 47]. In 2017, Mancarci et al. presented an integrated mouse single-cell RNA transcriptomics database, which proved the neuronal presence of P2X7 receptors [48]. Further studies confirmed the neuronal presence of P2X7R and showed that the roles of these receptors in the glia or neurons are not always mutually exclusive [49]. Here, we investigated P2X7R immunostaining signal following CSD and we have found an increase in the P2X7R signal in cortex, thalamus, striatum, hypothalamus and hippocampus following CSD in neurons, effecting both hemispheres, concordant with the neuroinflammation [41]. In addition, P2X7R antagonism thus blockage of the inflammatory cascade halted this increase in the neuronal cytoplasmic P2X7 signal in all of the aforementioned cortical and subcortical regions. Previous studies show that P2X receptors are internalized following increased extracellular ATP, as an adaptive response to protect the neurons from ATP-related toxicity [50]. This protective mechanism exists in various agonist-receptor combinations when high extracellular concentrations of the agonist has toxic effects to the cell [50, 51]. We hypothesize that the significant increase in extracellular ATP released by neurons during CSD binds to nearby neurons’ P2X7 receptors, leading to an increase in alarmins and cytokines. This increase further stimulates neuroinflammatory responses in astrocytes. The internalization of P2X7 receptors could be serving as an inherent protective mechanism activated by excessive P2X7R stimulation, aimed at mitigating the harmful effects of prolonged receptor activation. Moreover, we propose that blocking P2X7 receptors with an antagonist prevents this overstimulation, thus averting receptor internalization. Acute internalization of the P2X7 receptors, 20 min following the CSD, explains the increased cytoplasmic P2X7R signal. Meanwhile, P2X7R antagonist administration prevents this adaptive response, supporting our findings explaining the concordance between neuroinflammation and P2X7 signal increase following CSD [52]. Future studies that unravel the underlying mechanisms of these findings will enhance our comprehension of the purinergic signaling’s role in neuroinflammatory processes incited by CSD.

As the limitations of this study, we did not determine the sample sizes a priori and performed the experiments with anesthetized animals, which might be altering CSD characteristics. Furthermore, despite the suggested role of CSD in the pathophysiology of various disorders including stroke, subarachnoid hemorrhage and migraine [5, 53, 54], the relevance of CSD or its spread to subcortical structures to migraine symptomology is unclear. Despite the effect of CSD in inducing a behavioral phenotype that resembles migraine has been shown in mice [55], recent neuroimaging data suggest that aura does not initiate migraine attacks in humans [56]. Future studies should address whether CSD, or migraine aura, is an inciter for migraine headaches or it is only an epiphenomenon.

## Conclusion

In conclusion, this study shows that P2X7 receptors have an important role in neuroinflammation in cortical and subcortical structures (striatum, thalamus, hippocampus, hypothalamus) and in hypothalamic neuronal activation in both hemispheres following CSD. Moreover, P2X7R antagonism increased the latency of hypothalamic voltage deflection following CSD, pointing out the role of extracellular ATP as a mediator in the subcortical spread of CSD. CSD also caused an increase in the P2X7 signal that may be secondary to receptor internalization as an adaptive response to protect neurons from ATP-associated toxicity. These results suggest that P2X7 receptors could be a potential target to mitigate CSD-induced neuroinflammatory signaling.

## Methods

### Animals

In this study a total of 83 mice, including C57BL6, wild-type and/or transgenic mice (Thy1-ChR2), BALB/c and Swiss albino male/female mice, weighing 20–30 g were used. Standard housing conditions were applied, in 12 h light/dark cycles, at 22^o^C room temperature and ad libitum. All experiments were performed on a similar period of the day (in the morning) so as to avoid potential confounders. Animals were randomly chosen for experiments whereas s specific randomization sequence was not followed. All the experiments performed on laboratory animals are approved by Hacettepe University Animal Studies Ethical Committee (2008/55 − 6, 2010/49 − 1 and 2017/11 − 5 and 2023/41).

### Electrophysiology

#### Surgery

Before the surgical procedures, mice were anesthesized with xylazine (10 mg/kg, Alfazyne, 2% Alfasan, Netherlands) and Urethane (1.25 g/kg, Sigma-Aldrich, USA). Depth of the anesthesia was tested with a pinch to the paw and animals were transferred to the stereotaxic frame. During the experiments, animals were supplied with 2 L/min oxygen and followed up with spontaneous respiration. A rectal probe was used during the procedure and the body temperature was kept at 37±2^o^C with a homeothermic blanket. Meanwhile blood oxygen saturation was monitorized with a pulse oximetry.

After placing the mice in the stereotaxic frame, a skin incision was made under a stereomicroscope to expose bregma, parietal and frontal bones. A region on the posterior parietal bone was thinned using a drill for electrode placement. During drilling, cold saline solution was used to cool down the skull. Optogenetic laser stimulation was performed transcranially on the frontal bone (on motor cortex) while electrophysiological recording was made with an Ag-AgCl covered 1 mm pellet electrode on the posterior parietal bone. In the sham group the same surgical procedure was performed with wild-type mice, where CSD was never induced with laser stimulation. For all experiments, a single animal was used as the experimental unit.

### Optogenetic CSD threshold

Following the surgical procedures, the fiberoptic cable was placed on the frontal bone and optogenetic threshold experimental protocol was performed according to the method described in (Houben et al.). Laser source was adjusted to 4 mW using a photometer and 450 nm laser was applied step by step for 0.25; 0.5; 0.75; 1; 2; 3; 4; 5; 6; 7; 8; 9; 10; 11; 12; 13; 14; 15 s. After each stimulation, there was a wait of 5 min before the next stimulation to see if CSD is triggered where extracranial electrophysiological recordings were performed simulatenously. The energy transferred to the cortex was calculated as milijoules (mJ = mW x sec). After induction of CSD the experiment was terminated after 20 min. In threshold and subsequent immunohistochemistry experiments that are presented in this study, only a single CSD was induced and intracortical electrode insertion was not performed for these experiments.

### Subcortical electrophysiological recordings

So as to investigate the subcortical spread of the CSD, following opening a burr hole at the coordinates according to the bregma a tungsten electrode with a 1 μm tip diameter was placed to dentate gyrus (2 mm posterior, 1.5 mm lateral to bregma, 1.8 mm deep from the surface), to thalamus (ventral posterolateral nucleus, 1.6 mm posterior, 1.75 mm lateral to bregma, 3.25 mm in deep), to striatum (0.86 mm anterior, 1.5 mm lateral to bregma, 3 mm in deep), and to hypothalamus (anterior hypothalamic area, 1.46 mm posterior, 0.5 mm lateral, 5.5 mm in deep from the surface). After the placement of the electrode, there was a wait of 20 min before inducing CSD by pin-prick using a frontal burr hole. Electrophysiological recordings from a surface electrode was performed simultaneously. These experiments were performed both by optogenetic and the pin-prick CSD induction methods, due to the invasive nature of subcortical electrophysiological recordings. We did not observe a difference in the subcortical spread patterns of the CSD. Following optogenetic induction CSD, we observed its spread to the hypothalamus in six Thy1-ChR2 mice. We induced CSDs from the frontal cortex (over the skull) using 50 mA, 10 s of laser stimulation, at 15-minute intervals; and recorded spreading depolarizations from the hippocampus, thalamus, and hypothalamus. P2X7R antagonist or vehicle was administered before CSD induction during simultaneous hypothalamic and cortical electrophysiological recordings.

### Pharmacological agent

A highly potent (pK_i_:7.9±0.07), selective (EC_50_:78±19 ng.ml^− 1^) and BBB permeable P2X7R antagonist, JNJ-47965567 was used to investigate the effects of P2X7R antagonism on the subcortical spread of CSD and neuroinflammation. The pharmacokinetic properties of this drug was characterized in the literature indicating that the drug concentration peaks rapidly in the brain (in 15 min) and stays effective for 4–6 h [57]. Drug was freshly prepared for each experiment and dissolved in 30% solution of cyclodextrin-sulfobutyl ether sodium salt (vehicle), administered intraperitoneally 15 min before the CSD threshold protocol started.

### Immunostaining methods

Following the in vivo experiments, mice went under cardiac perfusion with 0.4% heparin followed by 4% paraformaldehyde (PFA). Only the mice that went under the less invasive optogenetic CSD induction approach were used for the immunofluorescent staining, to avoid the potential neuroinflammatory side effects of other induction methods. The brains were extracted and incubated in 4% PFA for 24 h then cryoprotected with 30% sucrose. Coronal sections of 20 μm were made using a cryostat and these sections were further used for immunostaining. Two different methods of immunostaining were used: indirect immunofluorescence, immunosignal hybridization chain reaction (isCHR),

### Indirect immunofluorescence

First, antigen retrieval was performed at 80^o^C for 10 min using citrate buffer (pH 6.0 and sections were washed with phosphate buffered saline (PBS) prior to blockage. Sections were blocked in room temperature with 10% normal goat serum (NGS)-0.5% Triton X-0.3 M glycine for an hour at room temperature. Then the sections were incubated with primary antibody of interest in the blockage solution (c-fos,1:200, Abcam/ab208942; NFκB-p65, 1:200, Cell Signaling Technology/8242; P2X7R, 1:50, Abcam/ab109054; S100β, 1:200, Abcam/ab52642; NeuN, 1:200, Millipore/MAB377) overnight at + 4^o^C. The day after sections were washed 3 times with PBS and incubated with secondary antibodies (Goat anti-rabbit Cy2, 1:200, Jackson Immunoresearch (JI)/111-225-144; Goat anti-rabbit Cy3, 1:200, JI /111-165-144; Goat anti-mouse Cy2, 1:200, JI /115-225-146; Goat anti-mouse Cy3, 1:200, JI /115-165-146) in blockage solution for 2 h in room temperature. After washing the sections with PBS three times, the sections were mounted with Hoechst-33,258 and imaged under confocal microscope.

### Immunosignal hybridization chain reaction (isHCR)

isHCR is a technique developed from fluorescent in situ hybridization (FISH), that amplifies the fluorescent signal using complementary primers which have fluorescent tags. This system was used with NFκB-p65 staining where spatial information is vital and signal is rather difficult to obtain. Following the incubation with the primary antibody the section was incubated with a secondary antibody that has a biotin tag. Using the strong affinity of streptavidin and biotin, streptavidin was used as a linker between the secondary antibody and the initiator primer sequence tagged with biotin. Then the amplifier sequences which have a fluorescent tag were used to amplify the signal. Background signal was reduced using graphene oxide. The details of the method is reviewed in [58].

### Analysis of the confocal images

Following NFκB-p65 and P2X7 staining, 2 images per hemisphere were taken for each brain region (cortex, striatum, hippocampus, hypothalamus, thalamus) per each animal. For analysis of the NFκB-p65 staining, total number of S100β positive cells (astrocytes) and the ones where NFκB-p65 was translocated to the nucleus were counted. For analysis of the P2X7R staining, cellular fluorescent intensity was investigated via ImageJ after the background was subtracted from each image. Then the colocalization of P2X7 with S100β or NeuN was made so as to determine the cellular subtype where the increase in the signal was observed. Following c-fos staining, positively-stained cells from thalamus, hypothalamus and cortex on each hemisphere were counted.

### Statistical analysis

The sample size for each experiment was stated in the legend and was not predetermined using statistical methods prior to experimentation. Sample sizes were based on previous studies in the field of migraine research. There is no set inclusion criteria for the experiments, although if an abnormality in the physiological parameters (body core temperature, blood SaO_2_) were to observed during the experiment, that animal was planned to be excluded from analysis. This exclusion criteria was never met and the data from all experiments were included in the analysis. The experimenters were not blinded. For the statistical analysis IBM SPSS 20 (Statistical Package for Social Sciences) and Graphpad Prism were used. When more than two independent group were compared ANOVA test or Kruskal-Wallis test was used decided upon the normal distribution parameters of the data. If the result was significant, appropriate post-hoc tests were used with Bonferroni correction. When normal distribution parameters were not met, Mann Whitney U test was used. The data that is presented in the bar graphs in mean with standard error of mean (SEM).

### Electronic supplementary material

Below is the link to the electronic supplementary material.

Supplementary Material 1

## Data Availability

The data that support the findings of this study are available from the corresponding author, upon reasonable request.
