# Supplementary material for: The effect of P2X7 antagonism on subcortical spread of optogenetically-triggered cortical spreading depression and neuroinflammation
Source: J Headache Pain. 2024 Jul 24;25(1):120. doi: 10.1186/s10194-024-01807-1 (PMC11267761; doi:10.1186/s10194-024-01807-1)
Supplement: Supplementary file 1 — Supplementary Material 1 [file 10194_2024_1807_MOESM1_ESM.docx]

**Supplementary Data**

**The Effect of P2X7 Antagonism on Subcortical Spread of Optogenetically-Trigged Cortical Spreading Depression and Neuroinflammation**

Burak Uzay, Buket Nebiye Demir, Sinem Yilmaz Ozcan, Emine Eren Kocak, Muge Yemisci, Yasemin Gursoy Ozdemir, Turgay Dalkara, Hulya Karatas


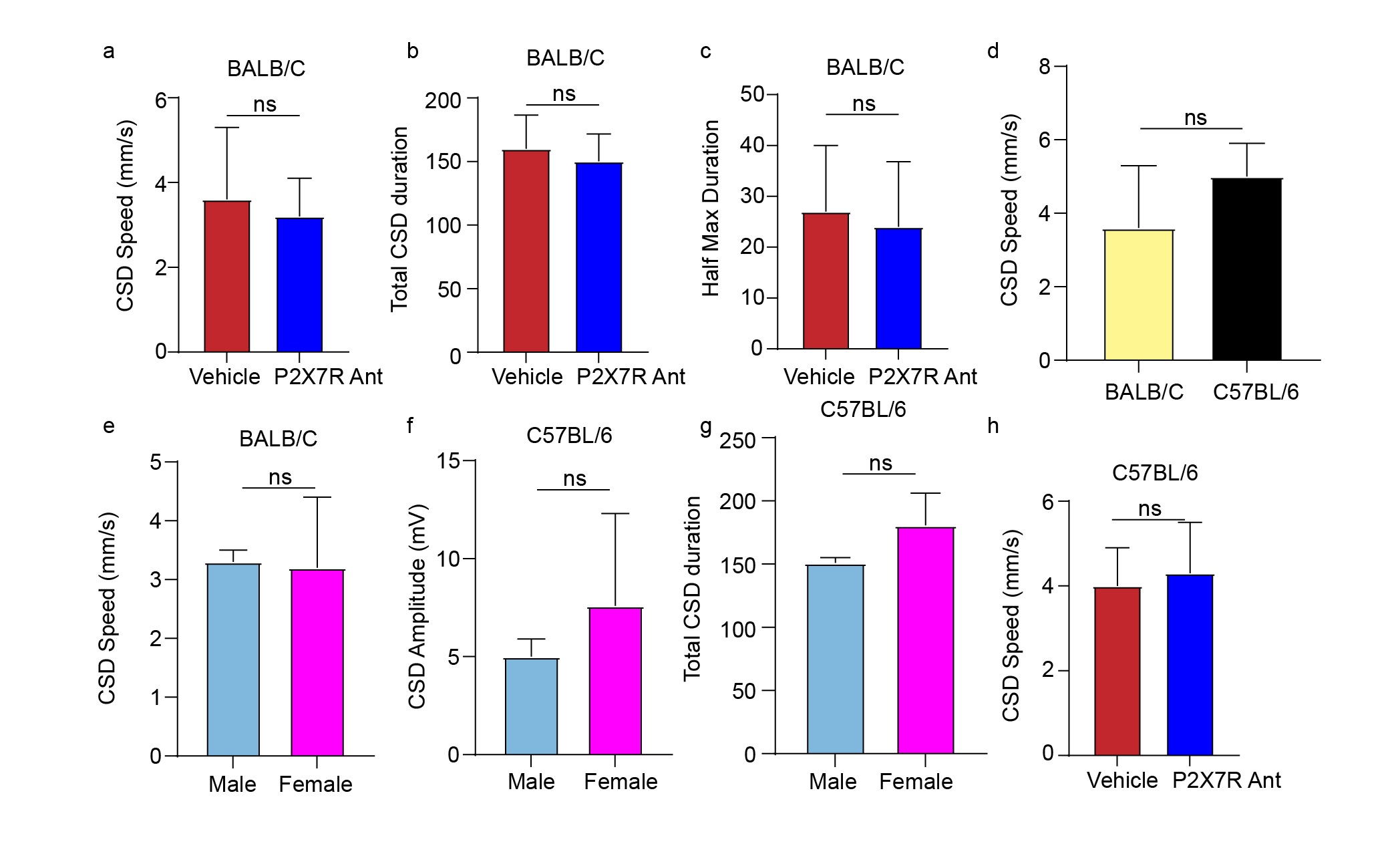


**Supplementary Figure 1.**a. CSD Speed in vehicle-treated and P2X7R antagonist-treated groups in Balb/C mice b. Total CSD duration in vehicle-treated and P2X7R antagonist-treated groups in Balb/C mice c. Half Maximum Duration of CSD in vehicle-treated and P2X7R antagonist-treated groups in Balb/C mice d. CSD Speed in Balb/C versus transgenic C57BL/6 mice e. CSD Speed in male versus female Balb/C mice f. CSD Amplitude in male versus female transgenic C57BL/6 mice g. Total CSD duration in male versus female transgenic C57BL/6 mice h. CSD Speed in vehicle-treated and P2X7R antagonist-treated groups in transgenic C57BL/6 mice (ns denotes non-significance, p>0.5)


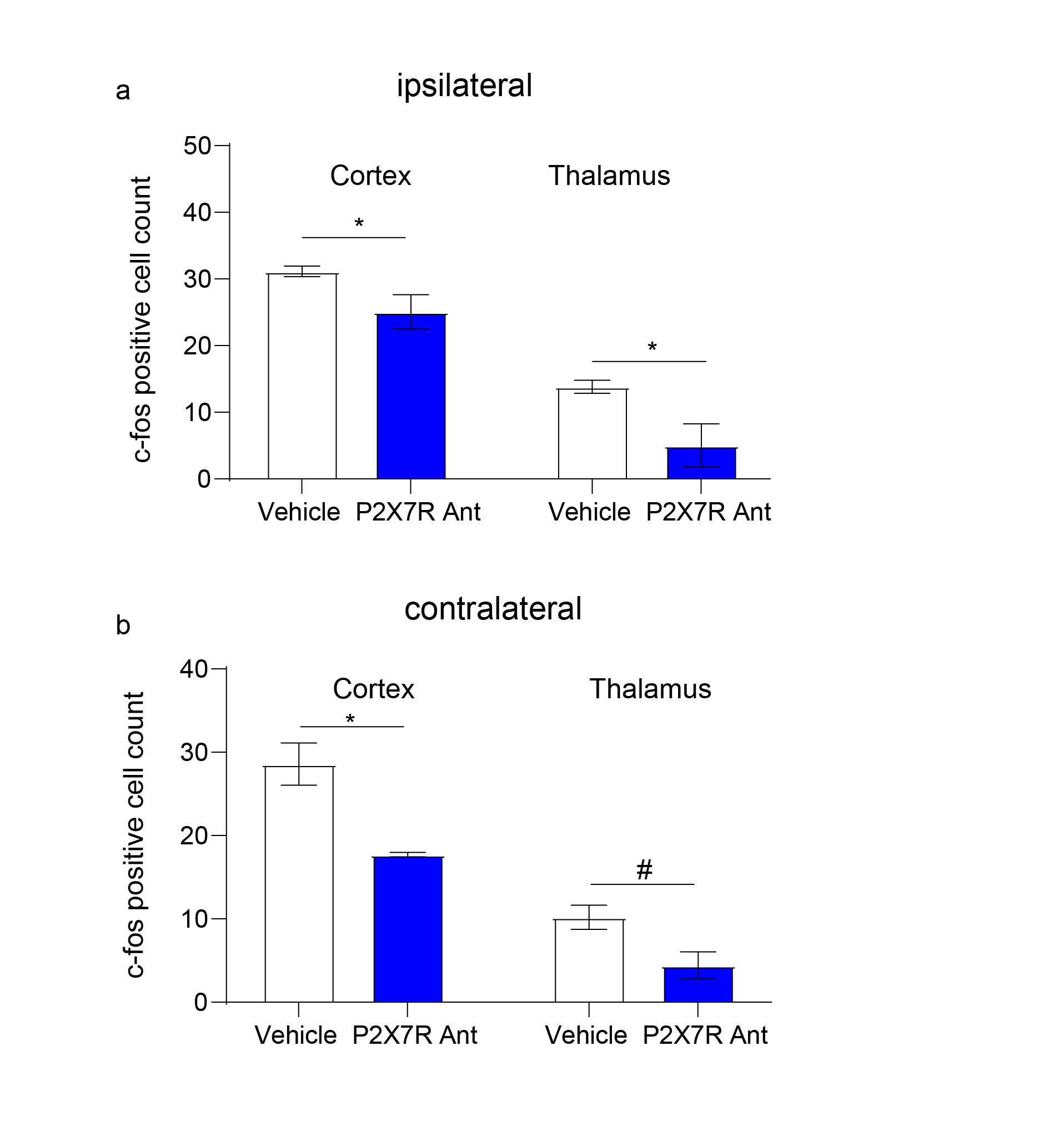


**Supplementary Figure 2** a. c-fos positive cell count in the ipsilateral cortex and thalamus following CSD preceded by vehicle or antagonist administration (p=0.03, p=0.02, respectively) b. c-fos positive cell count in the contralateral cortex and thalamus following CSD preceded by vehicle or antagonist administration (p=0.04, p=0.07, respectively) *(ns: p>0.05,#: p<0.1 *:p<0.05, **:p<0.01, ***:p<0.001)*


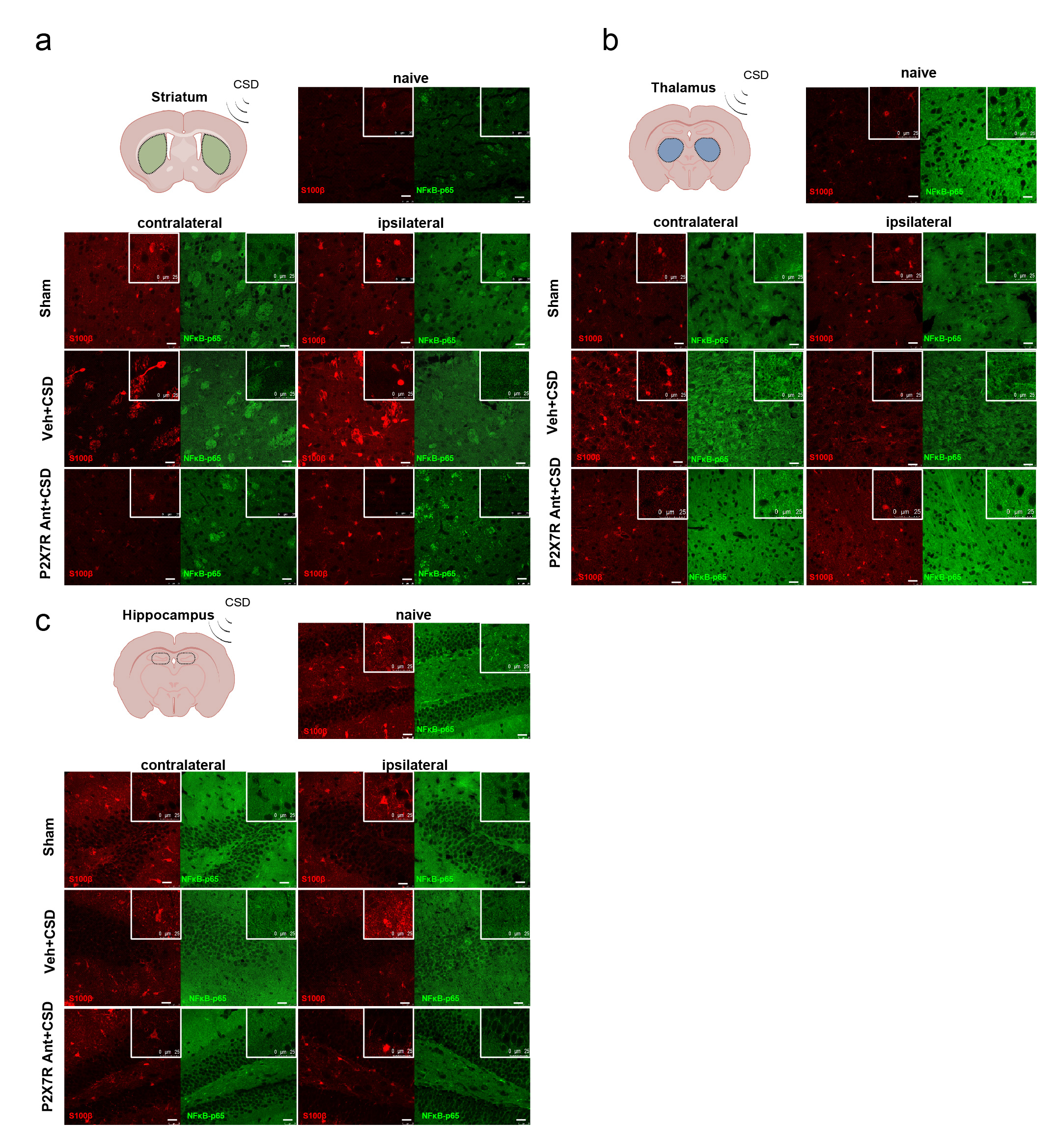


**Supplemental Figure 3 a.** Representative images of striatal NFκB-p65 and S100β immunofluorescent co-staining. *scale bar:25 μm* **b.** Representative images of thalamic NFκB-p65 and S100β immunofluorescent co-staining. *scale bar:25 μm* **c.** Representative images of hippocampal NFκB-p65 and S100β immunofluorescent co-staining. *scale bar:25 μm*


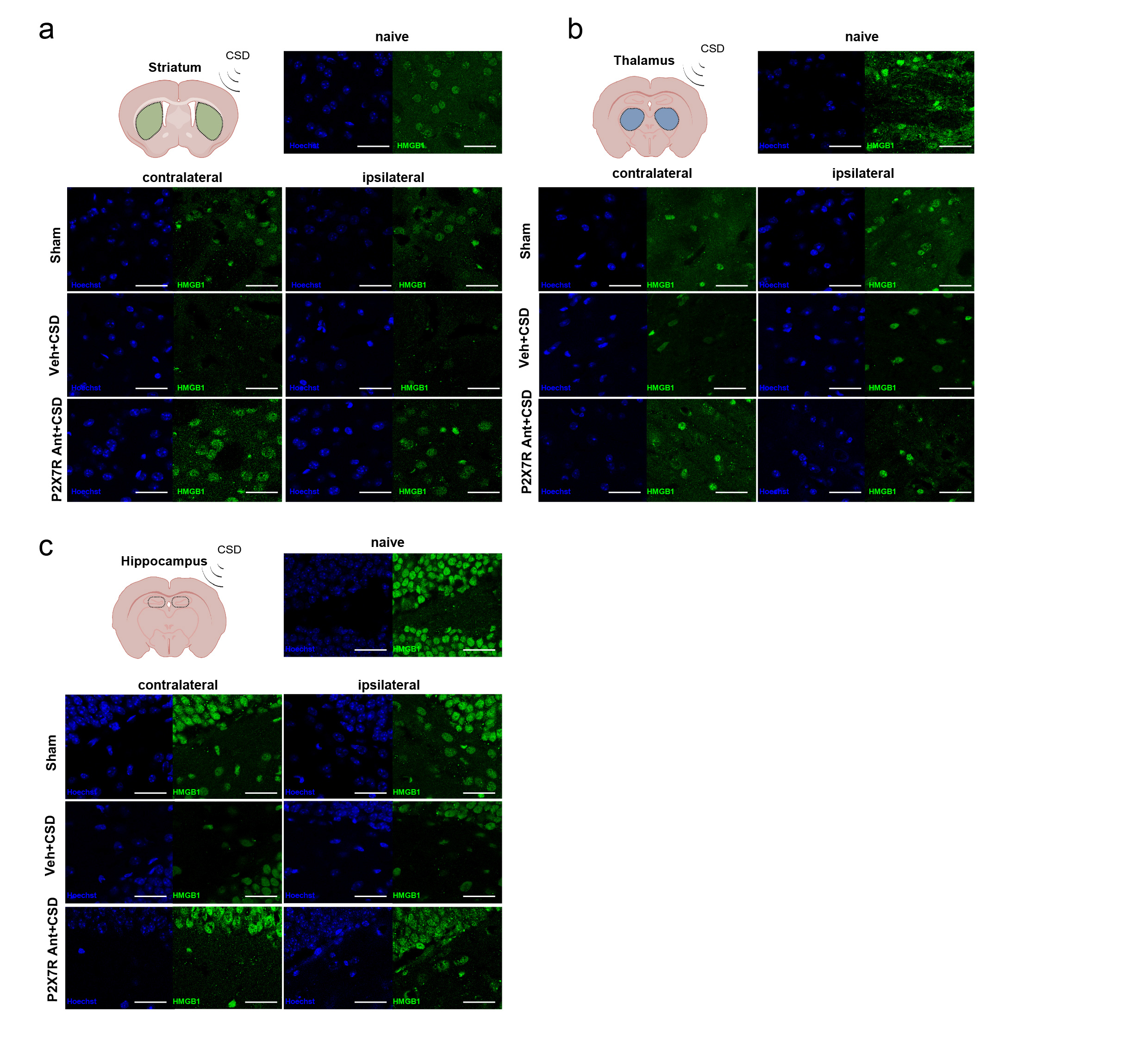


**Supplemental Figure 4 a.** Representative images of striatal HMGB1 immunofluorescent staining*.* *scale bar:25 μm* **b.** Representative images of thalamic HMGB1 immunofluorescent staining. *scale bar:25 μm* **c.** Representative images of hippocampal HMGB1 immunofluorescent staining. *scale bar:25 μm*


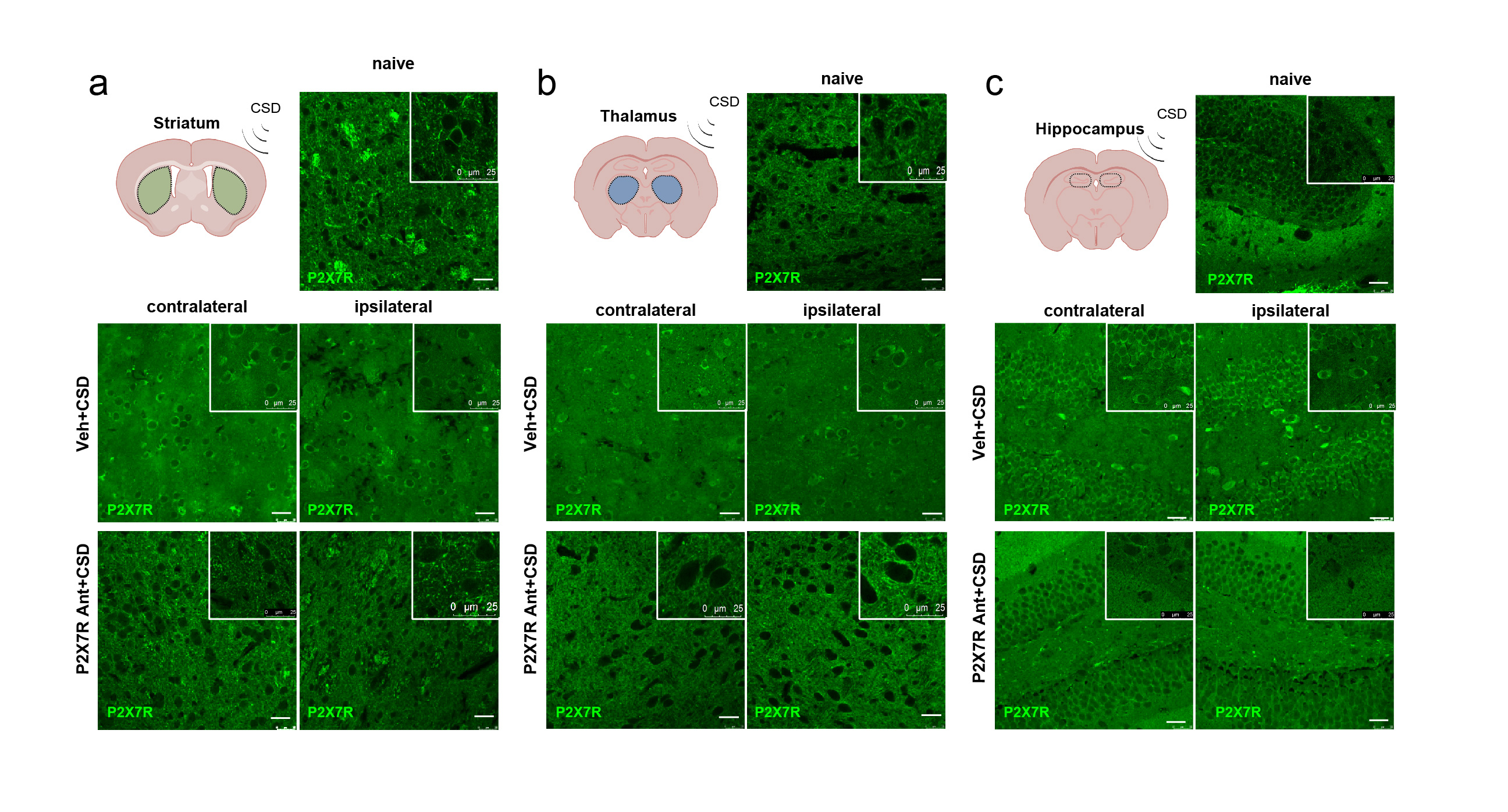


**Supplemental Figure 5 a.** Representative images of striatal P2X7R immunofluorescent staining. *scale bar:25 μm* **b.** Representative images of thalamic P2X7R immunofluorescent staining. *scale bar:25 μm* **c.** Representative images of hippocampal P2X7R immunofluorescent staining. *scale bar:25 μm*
